# GRAY DIVORCE: AN INCREASINGLY COMMON PATH TO UNCOUPLING IN LATER LIFE

**DOI:** 10.1093/geroni/igac059.153

**Published:** 2022-12-20

**Authors:** Torbjörn Bildtgård

## Abstract

Late life divorce is rapidly increasing in large parts of the Western world, in what has been described as a “gray divorce revolution”. In the US the incidence of gray divorce doubled between 1990 and 2010, in Sweden it has more than doubled since the millennium and in Israel it has almost doubled since 1996. Still, gray divorce remains rather invisible both in family sociology, which mainly focuses divorce at earlier ages, and in gerontology, which still tends to view widowhood as the single path to late life uncoupling. This symposium introduces current research on gray divorce and addresses a range of questions, such as: Why do people divorce late in life? How does gray divorce affect later life, including economy, health, support networks and relationships to adult children? How do cultural values shape gray divorce? What is the difference between his and her gray divorce? The presenters represent different countries (the US, Israel and Sweden) and approach the topic of gray divorce with both quantitative and qualitative data.

